# Least-Square-Method-Based Optimal Laser Spots Acquisition and Position in Cooperative Target Measurement

**DOI:** 10.3390/s22145110

**Published:** 2022-07-07

**Authors:** Kai Li, Feng Yuan, Yinghui Hu, Yongbin Du, Wei Chen, Chunyun Lan

**Affiliations:** 1 School of Instrumentation Science and Engineering, Harbin Institute of Technology, Harbin 150001, China; yuanf@hit.edu.cn (F.Y.); huyinghui@hit.edu.cn (Y.H.); 2 Beijing Institute of Aerospace Automatic Control, Beijing 100089, China; yiqijingdu@163.com (W.C.); sun_zhanghrb@163.com (C.L.)

**Keywords:** cooperative target, laser-projected spot, diode supply current, exposure time, least square method

## Abstract

The relative positioning precisions of coordinate points is an important indicator that affects the final accuracy in the visual measurement system of space cooperative targets. Many factors, such as measurement methods, environmental conditions, data processing principles and equipment parameters, are supposed to influence the cooperative target’s acquisition and determine the precision of the cooperative target’s position in a ground simulation experiment with laser projected spots on parallel screens. To overcome the precision insufficiencies of cooperative target measurement, the factors of the laser diode supply current and charge couple device (CCD) camera exposure time are studied in this article. On the hypothesis of the optimal experimental conditions, the state equations under the image coordinates’ system that describe the laser spot position’s variation are established. The novel optimizing method is proposed by taking laser spot position as state variables, diode supply current and exposure time as controllable variables, calculating the optimal controllable variables through intersecting the focal spot centroid line and the 3-D surface, and avoiding the inconvenience of solving nonlinear equations. The experiment based on the new algorithm shows that the optimal solution can guarantee the focal spot’s variation range in 5–10 pixels under image coordinates’ system equivalent to the space with a 3 m distance and 0.6–1.2 mm positioning accuracy.

## 1. Introduction

The position and attitude measurement of space targets have a wide range of application backgrounds in aviation, aerospace, satellite navigation and many other fields [1,2,3,4]. In these applications, in the implementation process of non-contact measurement, such as visual measurements, as the main measurement method, in order to meet the precise positioning of the target [5,6], the artificially set cooperative target is often used in cooperation with the shooting method, and the precise coordinates are obtained by extracting the center of the image value. This measurement method is often difficult to achieve further accuracy breakthroughs due to the long development cycle of the coordinate solution algorithm and complex data processing [7,8]. Moreover, the real-time requirements of coordinate solutions are in contradiction with the inherent nonlinearity of the solution formula. Therefore, the final position and attitude information need data compensation to be accurate [9], which means that the construction of a high-precision cooperative target measurement system requires the entire measurement system and data processing to be optimized. Traditional measurement conditions focus on the consideration of geometric quantities, including the relative position of the measuring instrument and the measured target, the indicators of flatness and straightness, and the definition of measurement error, while ignoring the consideration of other physical quantities that indirectly cause a change in spot position information. In this sense, the appropriate measurement conditions mostly include the selection of geometric quantities to be measured and the adjustment of physical parameters. Challenges still exist regarding how to optimize the parameters’ configuration and put them into practice [10,11,12]. Based on the above viewpoints, a ground simulation system [13,14] composed of two or more monocular planar array CCD cameras, a cooperative target of a cross-shaped target and paralleled screens can accurately reveal the process of space coordinates’ acquisition and position in three-dimensional space. In this ground simulation system, there are still challenges with improving the projected spot’s position precision regarding the calibration of the planar array CCD [15,16], efficiency of the spot centroid extraction algorithm [17,18], and optimization of the experimental conditions. Once the camera calibration method and centroid extraction algorithm are determined, the optimization of the experimental conditions will be the key point, the content of which involves the measuring principles, circumstances demand, measurement parameters selection and optimization, and the processing of measurement results.

In the ground simulation experiment, the quality of the measurement results depend on the choice of measurement conditions. The selection of new measurement conditions parameters, determination of parameter optimization criteria [19,20,21] and optimization algorithms [22,23,24,25,26] will determine the final accuracy and practicability of the ground simulation experiment.

The contributions of this paper are as follows: (1) Diode supply current and camera exposure time are adopted as two controllable parameters. (2) The novel state equation is established based on position information as state variables and physical parameters as controllable variables. (3) An innovative algorithm is proposed for determining the optimal controllable variables by calculating the intersection of line and surface. (4) Experiments based on the above algorithm are carried out to verify the accuracy.

The structure of the article is as follows: Related studies, prior knowledge and context are briefly introduced in Section 2. The principles and schemes including state equation under the image coordinates system, the description of the state variables and the optimization process are given in Section 3. Then, the optimized controllable variables are calculated by a novel algorithm. Section 4 introduces the algorithm validation experiment, provides the error curves, and an in-depth analysis of the calculation results is conducted. The conclusions are presented in Section 5.

## 2. Related Works

**Figure 1:**
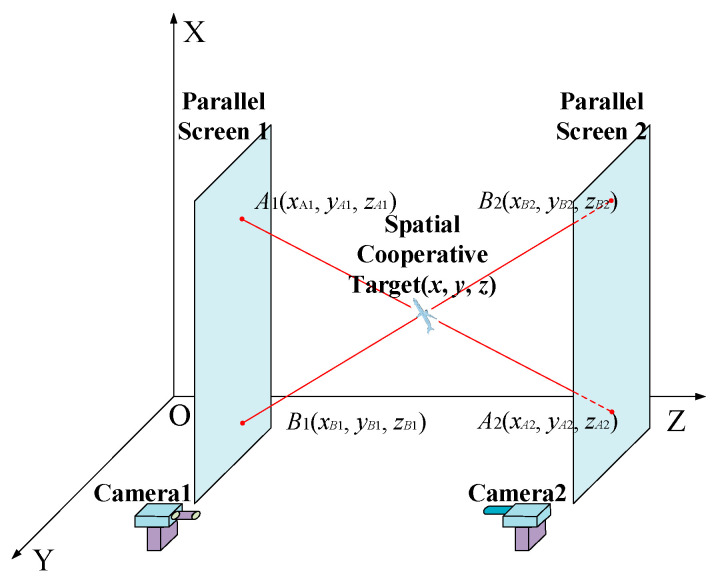
Schematic of space cooperative target measurement based on projected laser.

According to the geometric relationship of a straight-line intersection, the target coordinates can be calculated by the four projected points coordinates shown in Figure 1.

In Figure 1, the space target coordinates are calculated by the geometric location of the four projected spots: *A*_1_, *A*_2_, *B*_1_, and *B*_2_ on two parallel screens. The analytical solutions of the two laser trajectory line equations are expressed in Equation (1):

(1) $$\{\begin{array}{c} x=\frac{\left(\begin{array}{l} x_{A1}x_{B1}y_{A2}-x_{A2}x_{B1}y_{A1}-x_{A1}x_{B2}y_{A2}+x_{A2}x_{B2}y_{A1}\\ -x_{A1}x_{B1}y_{B2}+x_{A1}x_{B2}y_{B1}+x_{A2}x_{B1}y_{B2}-x_{A2}x_{B2}y_{B1} \end{array}\right)}{\left(\begin{array}{l} x_{A1}y_{B1}-x_{B1}y_{A1}-x_{A1}y_{B2}-x_{A2}x_{B1}\\ +x_{B1}y_{A2}+x_{B2}y_{A1}+x_{A2}y_{B2}-x_{B2}y_{A2} \end{array}\right)}\\ y=\frac{\left(\begin{array}{l} x_{A1}y_{A2}y_{B1}-x_{A2}y_{A1}y_{B1}-x_{A1}y_{A2}y_{B2}+x_{A2}y_{A1}y_{B2}\\ -x_{B1}x_{A1}y_{B2}+x_{B2}y_{A1}y_{B1}+x_{B1}y_{A2}y_{B2}-x_{B2}y_{A2}y_{B1} \end{array}\right)}{\left(\begin{array}{l} x_{A1}y_{B1}-x_{B1}y_{A1}-x_{A1}y_{B2}-x_{A2}x_{B1}\\ +x_{B1}y_{A2}+x_{B2}y_{A1}+x_{A2}y_{B2}-x_{B2}y_{A2} \end{array}\right)}\\ z=\frac{\left(\begin{array}{l} x_{A1}z_{A2}z_{B1}-x_{A2}z_{A1}z_{B1}-x_{A1}z_{A2}z_{B2}+x_{A2}z_{A1}z_{B2}\\ -x_{B1}z_{A1}z_{B2}+x_{B2}z_{A1}z_{B1}+x_{B1}z_{A2}z_{B2}-x_{B2}z_{A2}z_{B1} \end{array}\right)}{\left(\begin{array}{l} x_{A1}z_{B1}-x_{B1}z_{A1}-x_{A1}z_{B2}-x_{A2}z_{B1}\\ +x_{B1}z_{A2}+x_{B2}z_{A1}+x_{A2}z_{B2}-x_{B2}z_{A2} \end{array}\right)} \end{array}$$

Once the projected coordinates on the parallel screen under O-XYZ coordinate system—*A*_1_(*x_A_*_1_, *y_A_*_1_, *z_A_*_1_), *A*_2_(*x_A_*_2_, *y_A_*_2_, *z_B_*_2_), *B*_1_(*x_B_*_1_, *y_B_*_1_, *z_B_*_1_), and *B*_2_(*x_B_*_2_, *y_B_*_2_, *z_A_*_2_)—are determined, there is a unique space target position coordinate (*x*, *y*, *z*) corresponding to them. Mathematically, the accurate projected coordinates can be manifested as the centroid coordinates and the target precision depends on the precision of the projected coordinates [27,28].

### 2.1. Position Relationship between Cameras and Target Coordinates

**Figure 2:**
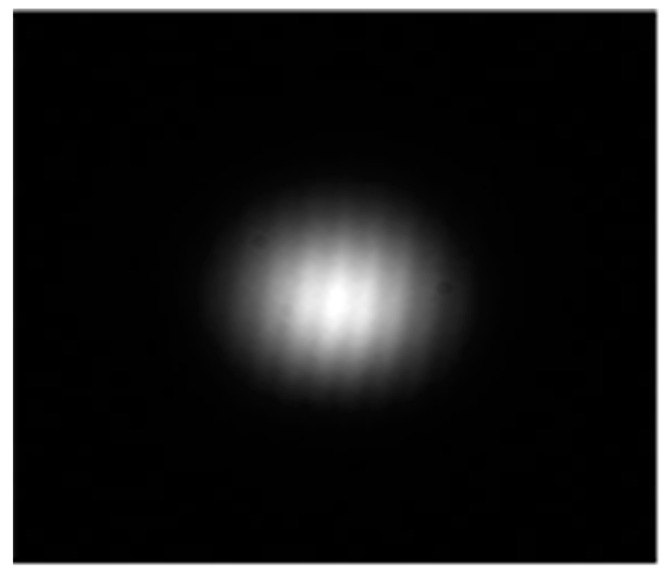
The gray level picture of a projected spot.

**Figure 3:**
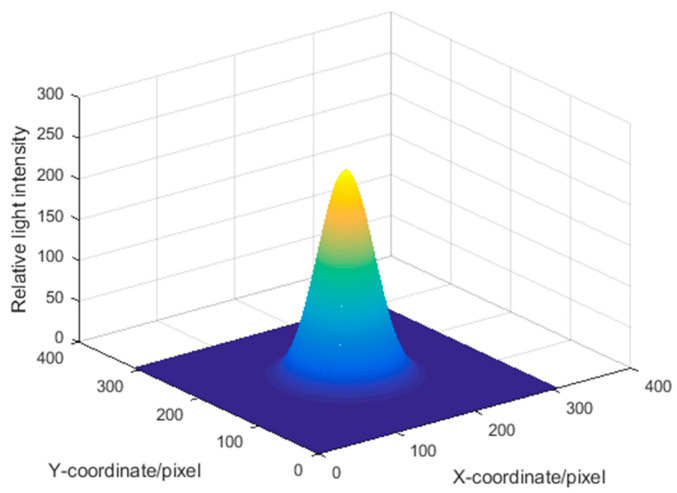
Light intensity distribution.

Assuming that each diode laser on the space target emits an ideal Gaussian beam, accordingly, the projected spots on the parallel screen express scattered spots, and the picture shot by the planar CCD camera shows grayscale images. Based on the above analysis, 2-D image coordinate axis is built in the plane of focal spot, in which the X and Y axes demonstrate the spot moving in a horizontal and vertical direction. The intensity distribution of the focal spot is described in Equation (2) [9], where *I*_0_ is the light intensity at the maximum value of the spot centroid, [*x*_0_, *y*_0_] is the spot centroid, and *D* is the spot diameter. The gray level and light intensity distribution are shown in Figure 2 and Figure 3, respectively:

(2) $$I\left(x,y\right)=I_{0}e^{\frac{-{\left(x-x_{0}\right)}^{2}-{\left(y-y_{0}\right)}^{2}}{{\left(\frac{D}{2}\right)}^{2}}}$$

**Figure 4:**
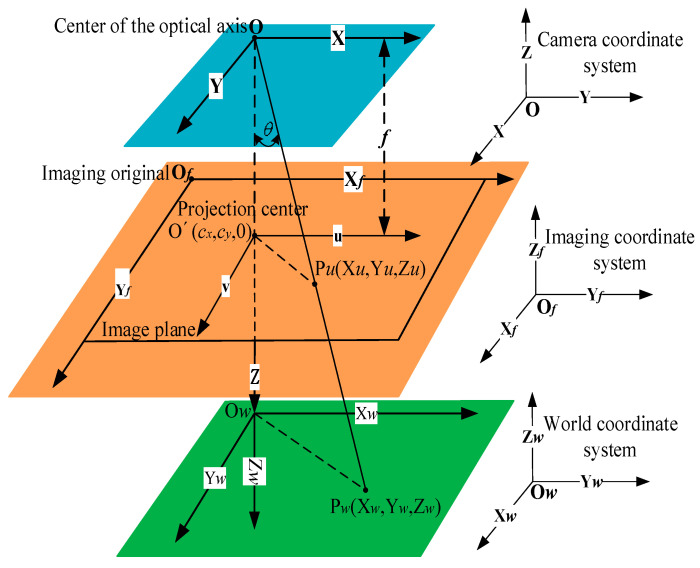
The diagram of a monocular camera under three coordinate systems.

The relationship between the three coordinate systems in monocular camera models are shown in Figure 4. The camera coordinate system (CCS) has the optical axis center of O as the origin. The plane created by the imaging origin O*f*, which is located upon the upper left of the plane, represents the imaging coordinate system (ICS). A real target coordinate system is a world coordinate system (WCS), the original center of which is O*w*.

The definition of each quantity expressed in Figure 4 is as follows: the coordinates of the target P under the WCS are set to be P*w* (X*w*, Y*w*, Z*w*), and the coordinates of its projection point under the ICS are P*u* (X*u*, Y*u*, Z*u*). *θ* is the angle between the connection line formed by the origin O and P*w* under the CCS and the optical axis Z, which represents the attitude information. *f* is the focal length of the camera. O′ is the intersection of the optical axis of the camera in the imaging plane and the projection of the origin O under the ICS, the coordinate value of which is (*c_x_*, *c_y_*, 0). 

If the physical size of each pixel is obtained by the calibrated internal parameters as *s_x_* = 1/*dx*, *s_y_* = 1/*dy*, the dimensions of which are 1/mm, the conversion formula, as shown in Equation (3) [29,30,31], is able to facilitate the coordinate transformation from ICS to CCS:

(3) $$\left(\begin{array}{c} u\\ v\\ 1 \end{array}\right)=\left(\begin{array}{ccc} s_{x} & 0 & c_{x}\\ 0 & s_{y} & c_{y}\\ 0 & 0 & 1 \end{array}\right)\left(\begin{array}{c} x\\ y\\ 1 \end{array}\right)$$

Followed by the matrix of ***R***_3×3_ and ***T***_3×1_, the transformation matrix from ICS to WCS is shown in Equation (4). If the inner parameters of [1/*s_x_*, 1/*s_y_*, *c_x_*, *c_y_ f*, *θ*] and the external parameters in ***R***_3×3_ and ***T***_3×1_ are obtained or calibrated, coordinate transformation is carried out:

(4) $$\left(\begin{array}{c} X_{u}\\ Y_{u}\\ Z_{u}\\ 1 \end{array}\right)=\left[\begin{array}{cc} R_{3\times 3} & T_{3\times 1}\\ 0_{1\times 3} & 1_{1\times 1} \end{array}\right]\left[\begin{array}{c} X_{w}\\ Y_{w}\\ Z_{w}\\ 1 \end{array}\right]$$

### 2.2. Laser Lighting Characteristics

As a stimulated radiation device, the semiconductor light-emitting diode (LED) is selected as the light source to meet the demands for space cooperative target measurement. To ensure supply current sustainability and light intensity, an efficient DC current regulator for the LED is needed. In this case, the LED is able to generate a controllable and high-quality light beam with a negligible optical distortion and tiny divergence angle. 

**Figure 5:**
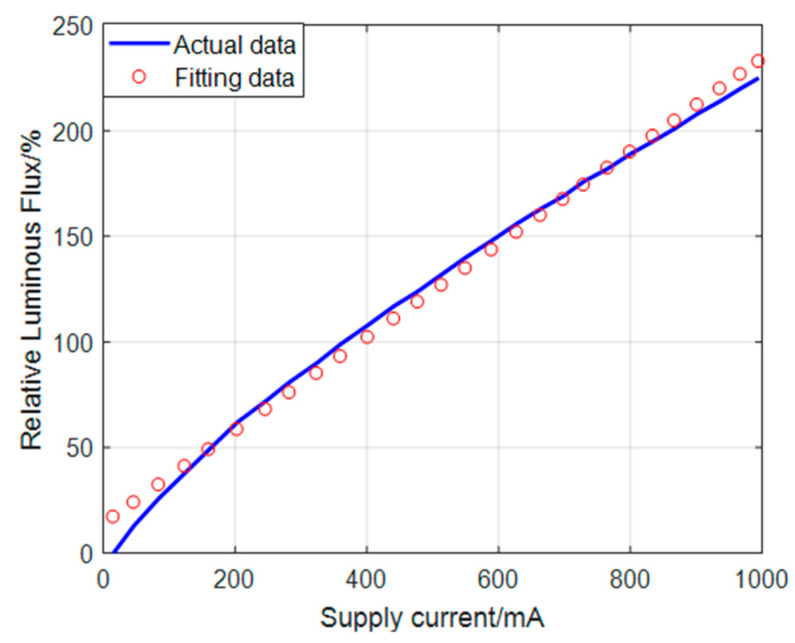
LED current vs. luminous flux.

In the CCS, the centroid algorithm is utilized to obtain the accurate position of the projected spot, and the change in the spot brightness has an effect on the position measurement. From a quantitative point of view, the relative luminous flux of the LED depends on the supply current. Figure 5 [13] shows this characteristic curve.

Figure 5 shows that the relative luminous flux is approximately proportional to the supply current in the special region where the supply current is between 100 mA and 400 mA and the relative luminous flux is less than 100%. The slope value of change is 0.22 based on the one-dimensional fitting data’s calculation. As the relative luminous flux and the illuminance have linear deterministic numerical correspondences, it can be considered that the illuminance and the supply current have a relationship as shown in Equation (5), where *I*_0_ has a similar definition and dimension as *I*_0_ in Equation (2), and *k* is 0.22 lx/mA:

(5) $$I_{0}=ki$$

### 2.3. Characteristics of the Camera’s Exposure Time

Ideally, the laser spot projected image is required to use the most suitable exposure time without saturating any of the pixels. This means that for the brightest pixels, the intensity is just below saturation. The approximate formula is shown in Equation (6) [18].

(6) $$H_{i}=\frac{1}{4}A\alpha RsT\tau \rho E_{s}{\left(\frac{D}{f}\right)}^{2}\frac{1}{{\left(1+m\right)}^{2}}t$$

In Equation (6): *H_i_*—the image gray level; *A*—camera gain factor; *α*—quantitative coefficient; *R*—the responsiveness of the CCD unit; *s*—the CCD unit area; *T*—optical lens transmittance; *τ*—the atmospheric transmittance; *ρ*—target reflection coefficient; *E_s_*—the target luminance; *D*—light flux aperture; *f*—focal length; *D*/*f*—relative aperture; *m*—imaging system magnification; *t*—the exposure time.

Based on the above analysis, Equation (5) shows that the supply current of the laser diode is proportional to the luminous flux within a specific interval, and Equation (6) shows that when the camera parameters are determined, the gray level of the image is approximately proportional to the exposure time. Therefore, adjusting the supply current and exposure time can effectively adjust the gray value of the spot-projected image, thereby adjusting the pixel coordinates of the spot centroid. 

## 3. Data and Method

**Figure 6:**
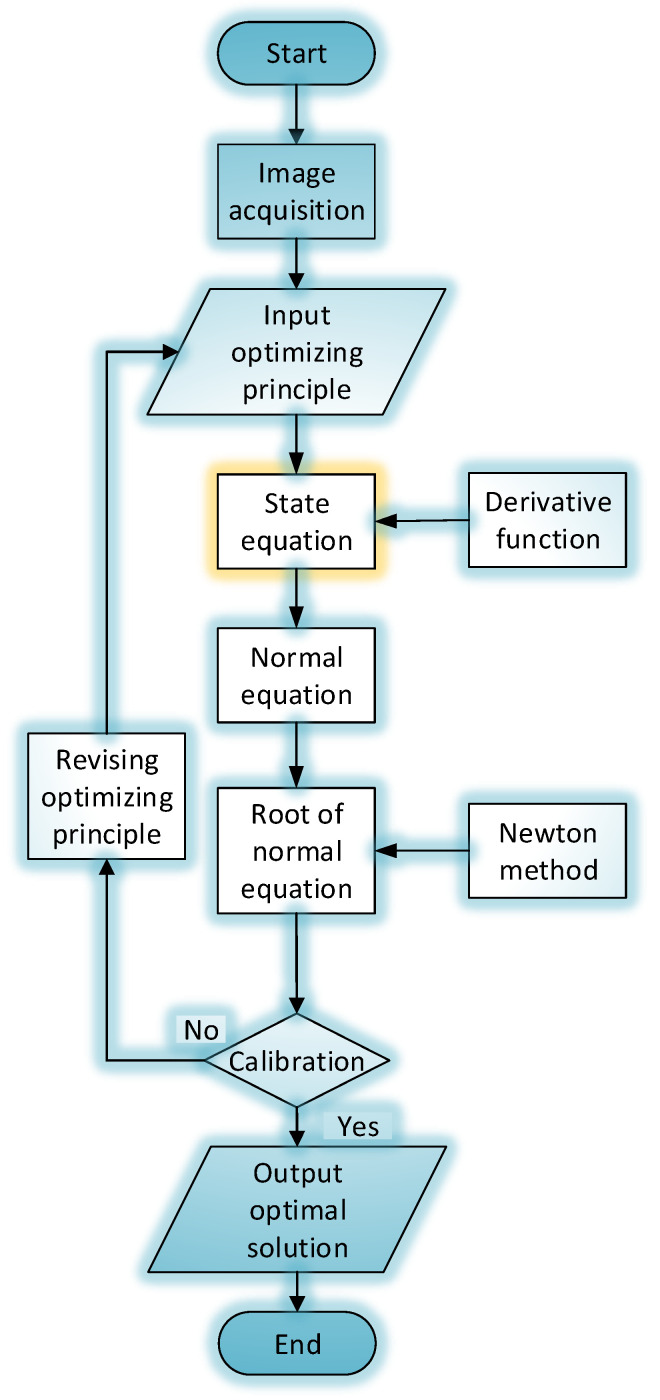
Data processing and optimizing flowchart.

The data processing and optimizing flowchart is displayed in Figure 6.

### 3.1. State Equation of the Imaging System

It can be concluded from Section 2.1 to Section 2.3 that the accuracy of the projected spot’s position depends on two factors, one of which is the coordinate transformation accuracy. For this factor, a calibration method, such as the Zhengyou Zhang Method [32], can determine accurate parameters, so the position precision can be guaranteed. The second factor is the brightness of the spot, which depends on two parameters: the supply current of *i* and the exposure time of *t*. For this part, the discrete state equation can comprehensively describe the process. 

The state equation is established as follows: First, the supply current of *i* and exposure time of *t* are implemented as controllable variables; Second, the laser-projected spot positions in the imaging plane under ICS are utilized as the fundamental state variables; Third, assuming the centroid coordinates of the la-ser spot is (*u*, *v*), the characteristics of which are essentially discrete variables, *k* indicates the camera frame rate and also characterizes the sample rate; therefore, the state equation can be built in Equation (7).

(7) $$\left[\begin{array}{c} u\left(k+1\right)\\ v\left(k+1\right) \end{array}\right]=\left[\begin{array}{c} u\left(k\right)\\ v\left(k\right) \end{array}\right]+\left[\begin{array}{c} \Delta u\left(k\right)\\ \Delta v\left(k\right) \end{array}\right]$$

In Equation (7), *u*(*k* + 1) and *u*(*k*), *v*(*k* + 1) and *v*(*k*) theoretically represent the same projected spot. Similarly, Δ*u*(*k*) and Δ*v*(*k*) show the position variation between two experimental conditions, respectively. This change in state variable has a functional relationship with the discretized supply current and exposure time. The characteristic expression is proposed in Equation (8) in the case that the variables of *i* and *t* are quantized with *k*:

(8) $$\left[\begin{array}{c} \Delta u\left(k\right)\\ \Delta v\left(k\right) \end{array}\right]=F\left[\begin{array}{c} i\left(k\right)\\ t\left(k\right) \end{array}\right]$$

### 3.2. Acquisition of Experimental Images 

**Figure 7:**
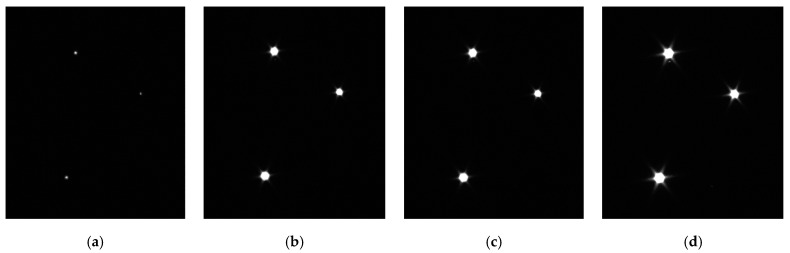
Laser spot images with different supply current (t = 500 ms). (**a**) Supply current = 0.12 A; (**b**) Supply current = 0.18 A; (**c**) Supply current = 0.35 A; (**d**) Supply current = 0.63 A.

**Figure 8:**
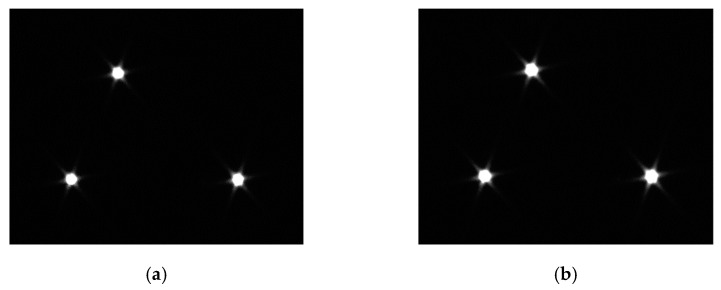

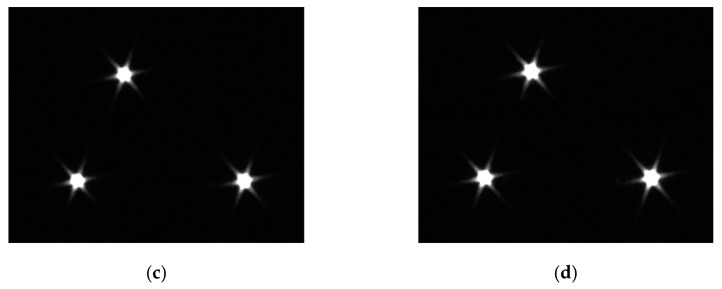
Laser spot images with a different exposure time. (i = 0.86 A) (**a**) Exposure time = 600 ms; (**b**) Exposure time = 1000 ms; (**c**) Exposure time = 1500 ms; (**d**) Exposure time = 2000 ms.

The details are as follows: three active light spots are implemented as substitutes for the projected spots to ensure the flatness of spots and equivalence of light characteristics. Three separated triangle-shaped lasers are settled in a fixed plate. In the first group, the supply current is regulated, then the CCD camera records the laser spot images; In the second group, the fixed plate rotates 180° counterclockwise, exposure time is adjusted, then a CCD camera records the laser spot images, the purpose of which is to avoid mutual interference between the two experimental conditions. Centroid coordinates of spots are calculated with the variation of *i* and *t*. Two groups of images are displayed in Figure 7 and Figure 8.

**Figure 9:**
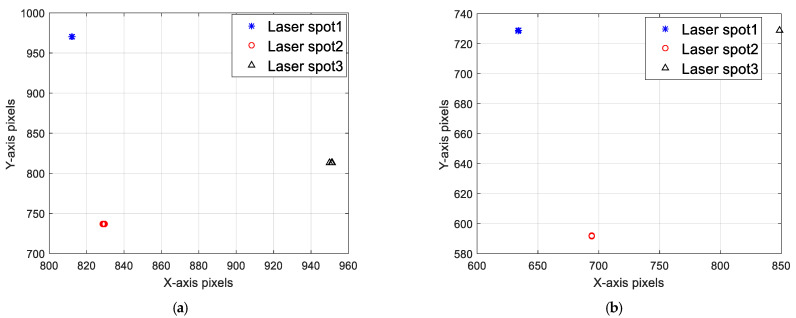
Distribution of laser spot centroid position. (**a**) Variation of supply current (t = 500 ms); (**b**) Variation of exposure time (i = 0.86 A).

In Figure 7 and Figure 8, the three projected spots are stable and simulate the stationary state of the actual space target point. The planar distribution of the projected spots’ centroid positions under two testing conditions is shown in Figure 9, in which different points and shapes represent three projected spots’ coordinates in the X–Y plane under ICS.

The coordinates shadow means that the actual projected spots’ position has a tiny perturbation. In fact, the absolute position of the three cooperative lasers remains stable during the experiment; therefore, the projected spots should completely overlap. Theoretically, the only changeable parameters are the supplied current of *i* and exposure time of *t*. The variation in the projected spot centroid positions is shown in Table 1 and Table 2, where the unit of the XY coordinates are pixels. The centroid is regarded as a reference point. The relationship between the area of the saturated pixel of the projected point and the corresponding parameters in the four sets of experiments are listed in Table 3 and Table 4.

On the basis of Equations (5) and (6), the macroscopic manifestation of the two factors is the position change in the centroid of the projected spots. As a result, the projected spot positions of (*u*, *v*) are regarded as state variables, then the supply current of *i* and the exposure time of *t* are considered as controllable variables, and an index function that satisfies the minimum position errors between the adjacent pixel points, need to be established. This method of modeling reveals guidelines for minimizing pixel point overlapping errors and avoids the phenomenon of non-correspondence due to the optimization of a single function. The optimized results of such an index function can be treated as the optimal controllable variables.

### 3.3. Optimization Process

The expression of Equation (8) indicates a decoupled relationship between the controllable variables of [*i*, *t*] and the state variable of [*u*, *v*]. Strictly speaking, there is a nonlinear functional connections with these variables. The optimization process attempts to investigate the independent impact factors of each controllable variable. Therefore, the index function is built in Equation (9), in which the product of the state vector and the transposed state vector signifies the dimensions of the second moment of the image:

(9) $${\left[\begin{array}{c} \Delta u\left(k\right)\\ \Delta v\left(k\right) \end{array}\right]}^{T}\left[\begin{array}{c} \Delta u\left(k\right)\\ \Delta v\left(k\right) \end{array}\right]=F^{T}\left[\begin{array}{c} i\left(k\right)\\ t\left(k\right) \end{array}\right]F\left[\begin{array}{c} i\left(k\right)\\ t\left(k\right) \end{array}\right]$$

When the space target point is stationary or moving slowly (*v* < 1 cm/s), the coordinates of the projected point in ICS should be stationary or moving slowly. In this sense, the only factors that affect the change in the coordinates of the projected spot centroid are *i* and *t*. The index function takes the partial derivative with respect to *i* and *t*, and the optimal parameter configuration can be obtained. Equation (8) is inherently a nonlinear equation by means of the simplification of Equations (5) and (6). Then, the mathematical problem of the partial derivative of the index function with respect to *i* and *t* can be transformed into the mathematical problem of the partial derivative of the index function with respect to *u* and *v*. The linearized algebraic equation of Equation (9) is shown in Equation (10). From this point of view, the projected spot position variation minimization is considered as the novel optimization criteria, and the results of optimizing process decide the optimal state variables. Based on the above analysis, the normal function and minimum criteria equation of Equation (10) is expressed in Equation (11). The optimal controllable parameter equation equivalent to the optimal state variable is shown in Equation (12):

(10) $$Q=\sum_{k=1}^{N}\left\{{\left[u_{j}^{*}\left(k\right)-u_{j}\left(k\right)\right]}^{2}+{\left[v_{j}^{*}\left(k\right)-v_{j}\left(k\right)\right]}^{2}\right\}$$

(11) $$\{\begin{array}{c} \frac{\partial Q}{\partial u}=0\\ \frac{\partial Q}{\partial v}=0\\ \frac{\partial^{2}Q}{\partial u^{2}}>0\\ \frac{\partial^{2}Q}{\partial v^{2}}>0 \end{array}$$

(12) $$\left[\begin{array}{c} i_{optimal}\\ t_{optimal} \end{array}\right]=F^{-1}\left[\begin{array}{c} u_{optimal}\\ v_{optimal} \end{array}\right]$$

In Equations (10)–(12): *N*—number of sampling images, in which *N* equals 4; u*_j_ (*k*), v*_j_ (*k*)—the coordinates of the optimal centroid position; *j*—label of each laser spot, in which *j* = 1, 2, 3; *Q*—novel optimization criteria; *i_optimal_*—optimal supply current; *t_optimal_*—optimal exposure time; *u_optimal_*—optimal horizontal pixel in ICS; *v_optimal_*—optimal vertical pixel in ICS. 

The optimization principles of Equation (10) are based on the criteria to satisfy the minimum value of the position residual’s square sum. Since *u* and *v* represent pixel values in two mutually orthogonal directions, there is no coupling between *u* and *v*. The optimizing procedures consist of three steps: First, the normal equation of Equation (10) is established, the form of which is the difference equation of Equation (10), and the displacement of *u*, *v* is with respect to *k*. Second, the solution of the normal equation is to be worked out, and the algorithm can be attributed to solve the pseudo-inverse matrix by Newton method. Third, it is recommended that the solutions are validated, and the precision of each solution needs to be determined. The final calculated results are listed in Table 5, in which the units are pixels.

It is confirmed from Table 1, Table 2 and Table 5 that the optimal value is equal to the arithmetic mean value of the experimental data in the same array. The results indicate that the two constraints conditions have been formally decoupled.

### 3.4. Determination of the Optimal Controllable Variables

**Figure 10:**
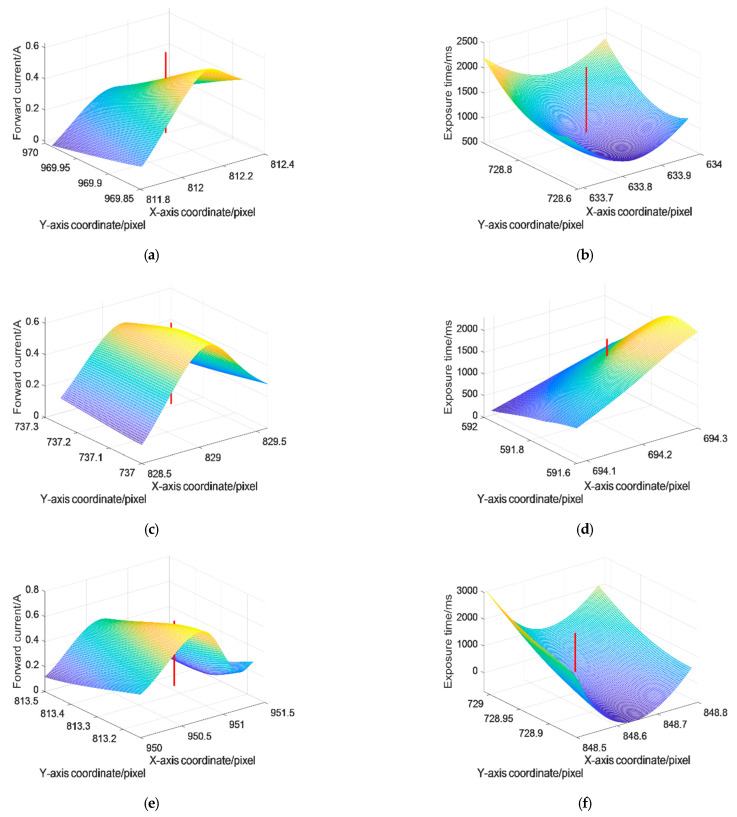
The optimal controllable variables selection of different lasers. (**a**) Laser spot 1 (supply current); (**b**) Laser spot 1 (exposure time); (**c**) Laser spot 2 (supply current); (**d**) Laser spot 2 (exposure time); (**e**) Laser spot 3 (supply current); (**f**) Laser spot 3 (exposure time).

Equation (7) reveals that there is essentially a nonlinear relationship between [*u*, *v*] and [*i*, *t*]. Equation (8) shows that in the local interval of [Δ*u*, Δ*v*], this nonlinear relationship can be approximately linearized according to Equations (5) and (6). In this case, the 3-D envelope surface can be drawn to describe this nonlinear relationship. The surface-extending trend shows that [*u*, *v*] continuously varies with *i* and *t* in the specified region. Furthermore, if the surface is projected onto the X–Z plane or the Y–Z plane, the curves of *u*-*i* or *v*-*i* and the curves of *t*-*u* or *t*-*v* can be obtained. Six surfaces in two groups of the experiment are shown in Figure 10, in which the left column expresses the relationship between *u*, *v* and *i*, and the right column expresses the relationship between *u*, *v* and *t*. 

In each of the subgraphs of Figure 10, four different sets of data are employed to generate each three-dimensional surface. The data derive from Table 1 and Table 2, and each group of data represents a set of controllable variables (*i*_1_, *i*_2_, *i*_3_, *i*_4_; *t*_1_, *t*_2_, *t*_3_, *t*_4_). On the basis of Equations (5) and (6), it is understood that *i* and *t* have a proportional relationship with *I*_0_ and *H*_i_, which means that the surface is analytic near the adjacent area of *u* and *v*. Due to its continuity, the linear polynomials fitting is able to approach the surface near the adjacent area of *u* and *v* in a certain precision. Once the optimal state variables of (*u*, *v*) are determined, the red straight line that characterizes the optimal variable can be formed, and the X–Y projected coordinates of the intersections between the straight line and the 3-D surface can be regarded as the best controllable variables of *i* and *t*. The coordinates of the intersection points can be calculated, as shown in Table 6.

## 4. Experimental Validation

### 4.1. Experiment Setup

**Figure 11:**
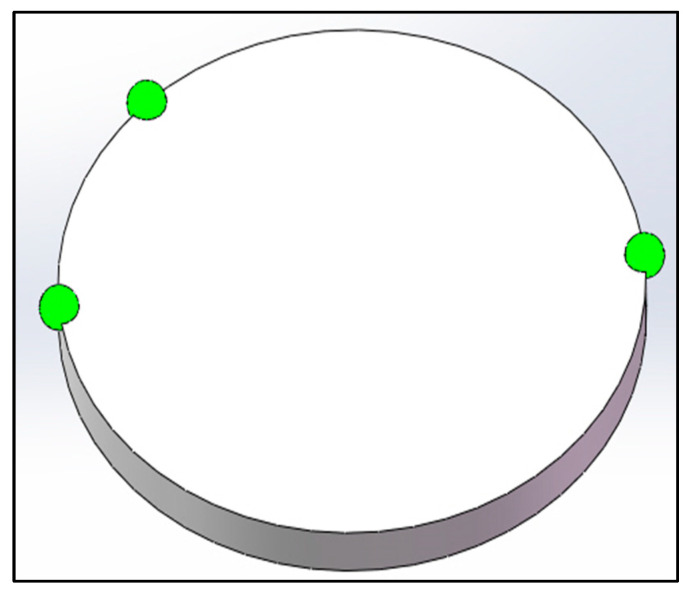
The schematic of target disc with three green LEDs.

In actual experiment, three active green LEDs were used as the laser sources to simulate the real reflected light and were mounted on a smooth disc as the coordinate target. Three LEDs form a right triangle, the aim of which is to ensure the accurate feature extraction of three landmark points after rotating 180°. The target disc schematic is shown in Figure 11.

**Figure 12:**
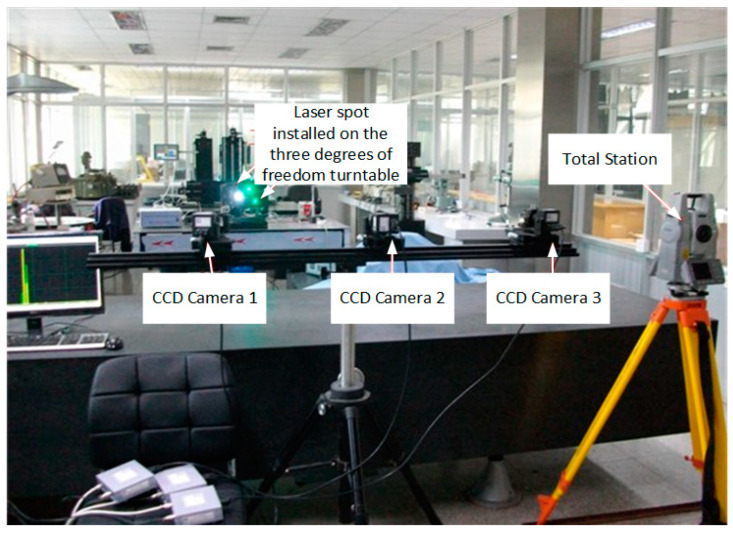
The on-site photos of the experimental platform.

Considering that the light spot of the LED laser simulates the projected light spot of the space cooperation target, the experimental scheme was adopted, in which the target disc was rotated 180° and the planar array CCD camera received images twice on one side. This can accurately simulate two images with a difference of 180° received by the same camera on one side, which is equivalent to two projected images of the same marker point received by two cameras on both sides. In the actual experiment, three CCD cameras were settled on a horizontal stent to record the three different laser spots, as shown in Figure 11, and three LEDs form a right triangle. Each laser spot has a unique current controller and separated exposure time switch. The on-site photo of the experimental platform is shown in Figure 12. 

### 4.2. Experiment Process and Data Analysis

**Figure 13:**
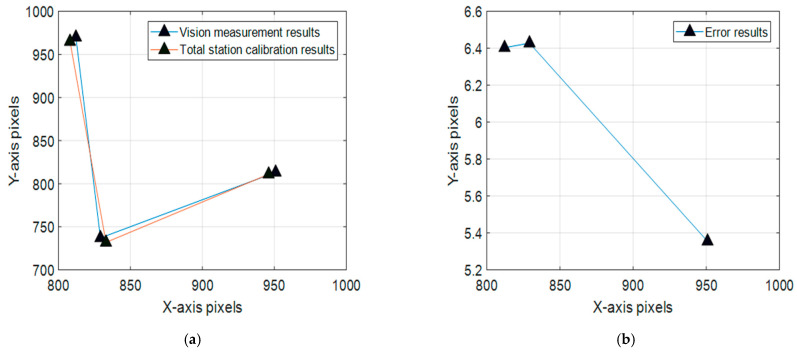

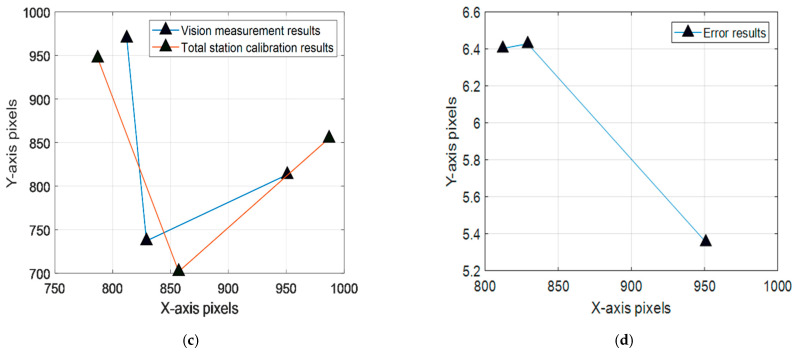
Calibration results and position error distribution. (**a**) Optimized results; (**b**) Optimized position error distribution; (**c**) Unoptimized results; (**d**) Unoptimized position error distribution.

The experiment was performed in an underground vibration isolation laboratory, and the light laser spots were installed on a three-axis turntable on a marble platform to simulate the cooperative space target, as shown in Figure 12. Three planar CCD cameras were installed to shoot the laser spot images and the images were transmitted into digital processing by high-speed data acquisition card and preprocessed on a computer. During the course of this experiment, the laser diode current and camera exposure time can be separately tuned by the calculated parameters, as shown in Table 5. The total station was used to calibrate the position of the LED spots, the differences between the vision measurement and total station are the errors, and the error curves are expressed in Figure 13.

Figure 13 reveals that the image pixel positioning errors of the optimized parameter configuration are an order of magnitude smaller than the image positioning errors of the unoptimized parameter configuration. From a quantitative analysis, the pixel errors can approach in 5–10 pixels, which means the position precision can satisfy 0.6–1.2 mm with a 3 m distance and 55 mm focal length through equivalent conversion. The comparative tests show that the ordinary experiment without variables’ optimization can only reach 30–50 pixels, which means the position precision can satisfy 3.6–6 mm with a 3 m distance. The positioning precision of the cooperative target position was improved.

The type and optical parameters of the cameras and lenses are listed in Table 7. For the experiment, a series of zoom lenses were chosen to ensure a certain depth of field and ensure a clear image, the internal parameters of which were calibrated in advance.

**Figure 14:**
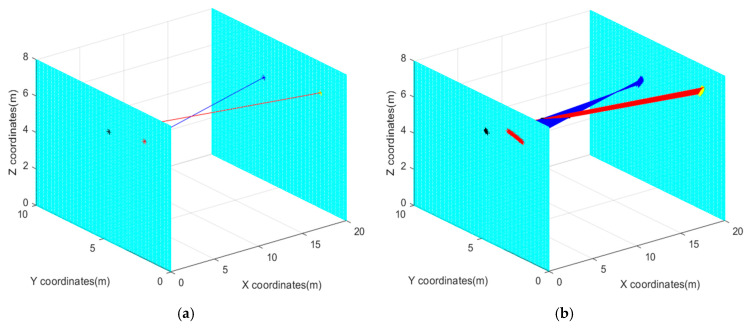
Comparative simulation results (**a**) Optimized simulation curves; (**b**) Unoptimized simulation curves.

If the field of view distance is enlarged to 10 m, and the distance between parallel screens is equal to 20 m, the calculation, which is based on the above analysis, reveals that the spot positioning error without using the optimized controllable parameters continues to be amplified, the simulation curves of which are shown in Figure 14. 

Figure 14 shows that when the field of view is enlarged, due to the magnification of the distance and cumulative effect of errors, the final positioning error is converted to the space target coordinate point will be on the order of 0.1–1 m, which seriously affects the reliability and stability of the measurement results. However, if the optimized control parameters are employed, the positioning error can be kept on the order of centimeters, and the measurement precision of a certain index can still be guaranteed.

## 5. Conclusions

In this work, the space cooperation target position measurement experiment simulated by LED laser point projected is implemented. The variables of supply current *i* and exposure time *t* are the two key factors that influence the position precision of XY coordinates in the ICS. The nonlinearities and 3-D surface, which are able to characterize the functional relationship between (*u*, *v*) and *i* or *t*, are calculated. This novel idea is proposed by the repeated spot position of (*u*, *v*) as state variables, taking the controllable variables of *i* and *t* as optimizing variables, and acquiring the optimal controllable values by LSM. The results of this new method are validated by our experiment, which is essential for satisfying the optimal measurement conditions. The conclusion can be drawn that the variables of supply current and exposure time can be separately adjusted within the controllable range in spite of the nonlinear relationship, and this causes the two variables to simultaneously reach their optimal values. The experimental results show that this new method can effectively improve the positioning precision of the light spot in the image coordinate system within a certain field of view. After conversion, it can effectively improve its positioning accuracy in the world coordinate system.

This work still needs to be improved in three aspects: First, the premise of the experiment is that the space target is stationary or moving slowly (*v* < 1 cm/s), and if the object moves quickly, how to accurately describe the influence of control parameters on the position accuracy will be worthy of further discussion. Second, the LED light spot is used to simulate the real projected spot, and the area array CCD is used to shoot the target rotated 180° to one side to simulate the real dual-screen shooting. The influence of these factors on measurement accuracy is worthy of further study. Third, other influencing factors aside from *i* and *t* must be fully discussed for space-cooperative coordinate measurements. For the above three aspects, the theoretical derivation and experiments may also predict other factors that can affect accuracy, which may be parameter-controlled and optimized using such methods to achieve a higher position accuracy.

## Figures and Tables

**Table 1:** Laser spot centroid position (x, y) of various supply current.

Supply Current (A)	Laser 1 (Pixel)	Laser 2 (Pixel)	Laser 3 (Pixel)
0.12	(811.81, 969.86)	(828.50, 737.00)	(950.00, 813.50)
0.18	(812.29, 969.99)	(829.60, 737.25)	(951.18, 813.24)
0.35	(812.19, 969.94)	(829.33, 737.19)	(951.36, 813.13)
0.63	(812.13, 969.85)	(829.11, 737.10)	(950.84, 813.22)

**Table 2:** Laser spot centroid position (x, y) of various exposure time.

Exposure Time (ms)	Laser 1 (Pixel)	Laser 2 (Pixel)	Laser 3 (Pixel)
600	(633.85, 728.64)	(694.08, 591.74)	(848.58, 728.96)
1000	(634.00, 728.72)	(694.30, 591.97)	(848.51, 728.86)
1500	(633.88, 728.92)	(694.19, 591.60)	(848.51, 728.86)
2000	(633.69, 728.89)	(694.23, 591.71)	(848.78, 729.01)

**Table 3:** Area of saturated pixels with different supply current.

Supply Current (A)	Laser 1 (Pixel^2^)	Laser 2 (Pixel^2^)	Laser 3 (Pixel^2^)
0.12	13	13	4.5
0.18	130	127	84
0.35	149.5	130	86
0.63	235.5	212.5	159.5

**Table 4:** Area of saturated pixels with different exposure time.

Exposure Time (ms)	Laser 1 (Pixel^2^)	Laser 2 (Pixel^2^)	Laser 3 (Pixel^2^)
600	116.5	131	134.5
1000	157.5	176	185.5
1500	225.5	229.5	241
2000	267	296.5	308.5

**Table 5:** The optimal (x, y) values with different conditions.

Results	Laser 1 (Pixel)	Laser 2 (Pixel)	Laser 3 (Pixel)
Supply current	(812.11, 969.91)	(829.14, 737.14)	(950.85, 813.27)
Exposure time	(633.85, 728.79)	(694.20, 591.76)	(848.60, 728.92)

**Table 6:** Calculation results.

Characteristics	Laser 1	Laser 2	Laser 3
Supply current (A)	0.47	0.60	0.58
Exposure time (ms)	734	1610	997

**Table 7:** Camera and lens optical parameters.

Camera Type	Resolution	Optical Size	Pixel Size	Frame Frequency	A/D Transfer Precision	Pixel Depth	Exposure Style	Shutter Time	Laser Wavelength	Field of View Distance
MER-500-7UM-L	2592 × 1944	1/2.5 inch	2.2 µm × 2.2 µm	7 fps	12 bit	8 bit	ERS/GRR	6 µs–1 s	480–550 nm	3 m
